# Supplementary material for: Circulating cell-free DNA methylation patterns indicate cellular sources of allograft injury after liver transplant
Source: Nat Commun. 2025 Jun 17;16:5310. doi: 10.1038/s41467-025-60507-9 (PMC12174327; doi:10.1038/s41467-025-60507-9)
Supplement: Supplementary file 1 — Supplementary Information [file 41467_2025_60507_MOESM1_ESM.pdf]

Supplementary Information

Supplement to:

"Circulating cell-free DNA methylation patterns indicate cellular sources of allograft injury after liver transplant." McNamara *et al.* 2025.

Contents:

Supplementary Figures and Legends

Supplementary Notes

Supplementary References

Supplementary Figure 1

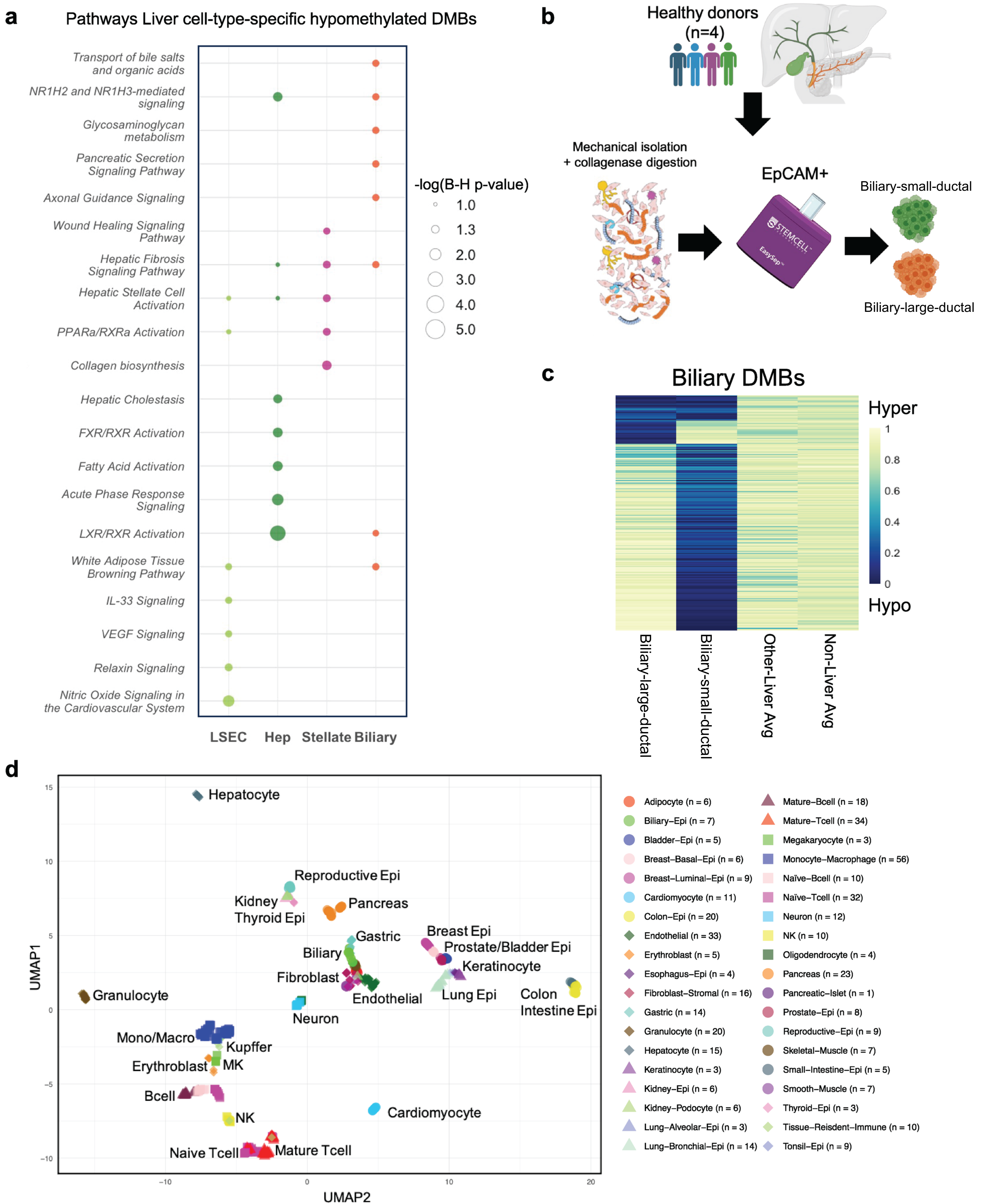

Supplementary Figures and Legends

Supplementary Figure 1. Characterization of human healthy cell-type-specific reference methylation data. a, Significant pathways related to the biological function of genes annotated to liver cell-type-specific hypomethylated blocks. b, Digestion of biliary tissues and enrichment of EpCAM (+) epithelial populations from the small-ductal versus large-ductal epithelial layers. c, Heatmap of biliary epithelial DMBs. Each cell shows the average methylation across all CpGs in the block comparing biliary-large-ductal, biliary-small-ductal, average across other liver cell-types, and average across all non-liver cell-types in the atlas. d, UMAP projection depicting relationship between different cell types with WGBS reference datasets included for analysis. Average methylation was calculated for each sample within blocks of at least three CpG sites and the top 10% of captured blocks were selected showing the highest variability across all samples. Figure 1b created in BioRender (2025) [https://BioRender.com/r28h425].

Supplementary Figure 2

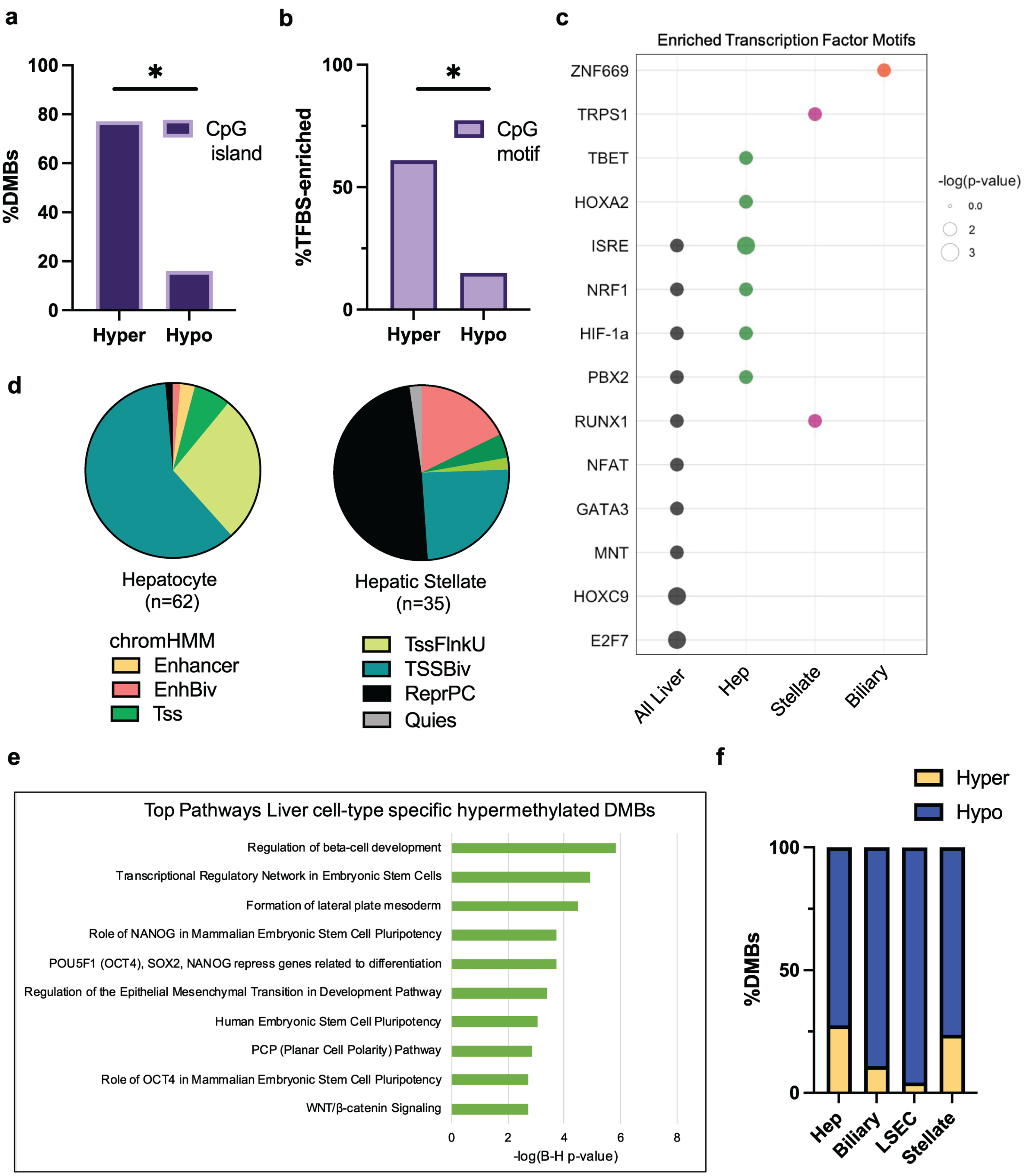

Supplementary Figure 2. Characterization of liver cell-type-specific hypermethylated DNA blocks. a, Percent of liver cell-type-specific blocks that overlap with CpG islands based on UCSC hg19 annotations (77% hypermethylated; 16% hypomethylated blocks) (Fisher's exact test, two-sided, p=0.0001). b, Percent of motifs enriched in liver cell-type-specific blocks containing a CpG dinucleotide (61% hypermethylated; 15% hypomethylated) (Fisher's exact test, two-sided, p=0.0005). c, Top TF binding sites enriched within liver cell type-specific hypermethylated blocks, from HOMER known motif analysis. Captured blocks without liver cell type-specific methylation were used as background. d, Fraction of cell type-specific hypermethylated blocks labeled as different chromatin states in chromHMM annotations from the same cell-type. e, Top 10 pathways related to the biological function of genes annotated to liver cell-type-specific hypermethylated blocks. f, Percent of hyper- and hypo-methylated DMBs identified for each liver cell type.

Supplementary Figure 3

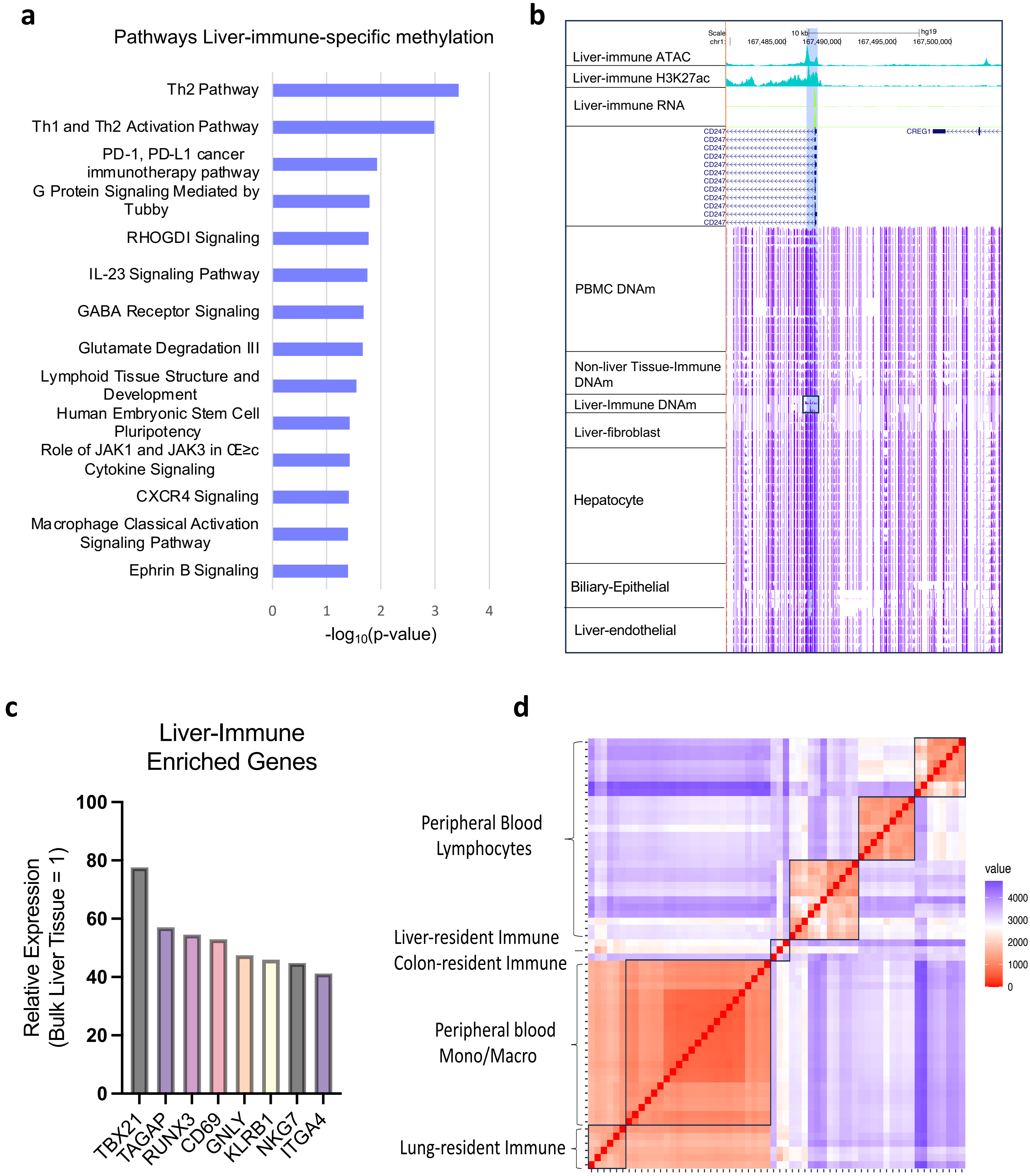

Supplementary Figure 3. Characterization of liver-resident immune cell-type-specific DNA methylation. a, Significant pathways related to the biological function of genes annotated to liver-immune cell-type-specific hypomethylated blocks. b, Example of one liver-immune-specific hypomethylated block (highlighted in blue), within the *CD247* gene locus and upstream of *CREG1* that are both highly expressed in liver-resident immune cells (green track). The alignment from the UCSC genome browser depicts the average DNA methylation (DNAm, purple tracks) across WGBS samples from five different liver cell-types as well as PBMC samples. Chromatin organization marks in liver-resident immune cells are displayed (blue tracks) to show accessibility (ATAC-seq) and regulatory function (H3K27ac binding). c, Enriched expression of liver-resident-immune genes (over 40-fold) relative to bulk liver tissue to validate purity and identity of starting cell populations. In addition to enriched expression of immune cell gene expression programs (TBX21, TAGAP, RUNX3, CD69), we also observed enrichment of several liver-resident lymphocyte and MAIT-Tcell genes (GNLY, KLRB1, NKG7). d, Distance heatmap showing similarity of reference methylomes. Liver-resident immune cell methylomes cluster distinctly and are most similar to colon tissue-resident macrophage methylomes in between peripheral blood macrophages and peripheral blood lymphocyte methylomes. Average methylation was calculated for each sample within blocks of at least three CpG sites and the top 10% of captured blocks were selected showing the highest variability across all samples.

Supplementary Figure 4

a

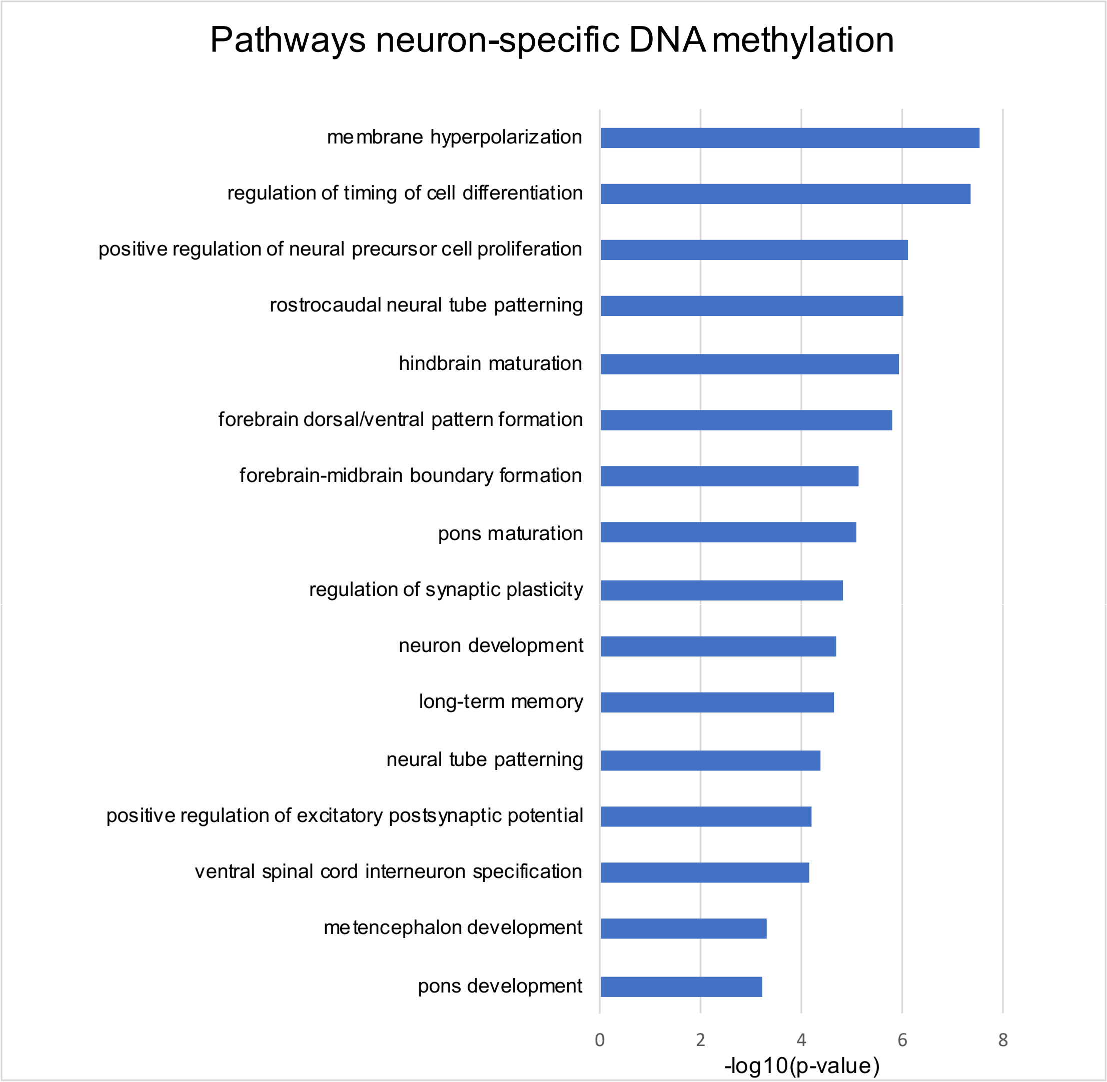

b

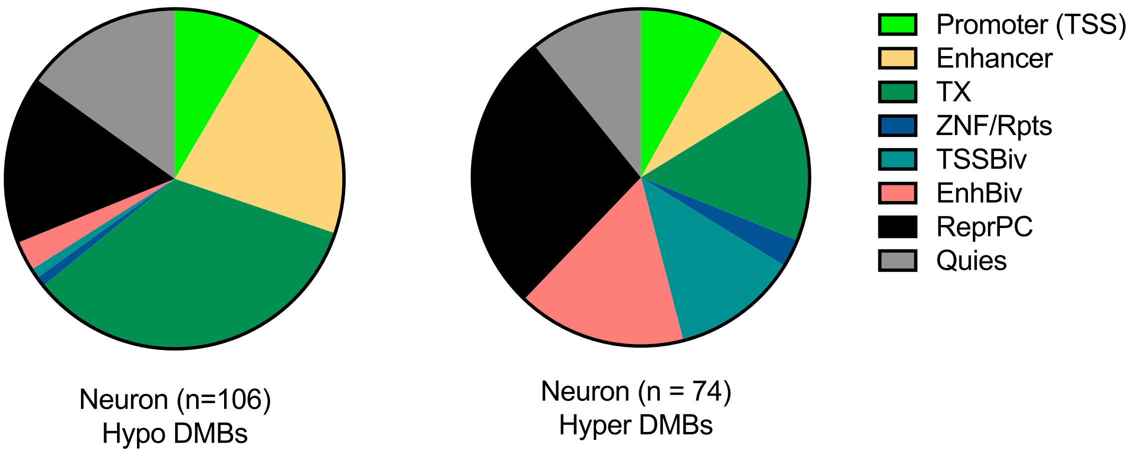

Supplementary Figure 4. Characterization of neuron-specific DNA methylation. a, Significant pathways related to the biological function of genes annotated to neuron cell-type-specific hypomethylated blocks b, Fraction of neuron-specific DNA methylation blocks labeled as different chromatin states in chromHMM annotations from the same cell-type (downloaded from the ENCODE project ENCSR539JGB).

Supplementary Figure 5

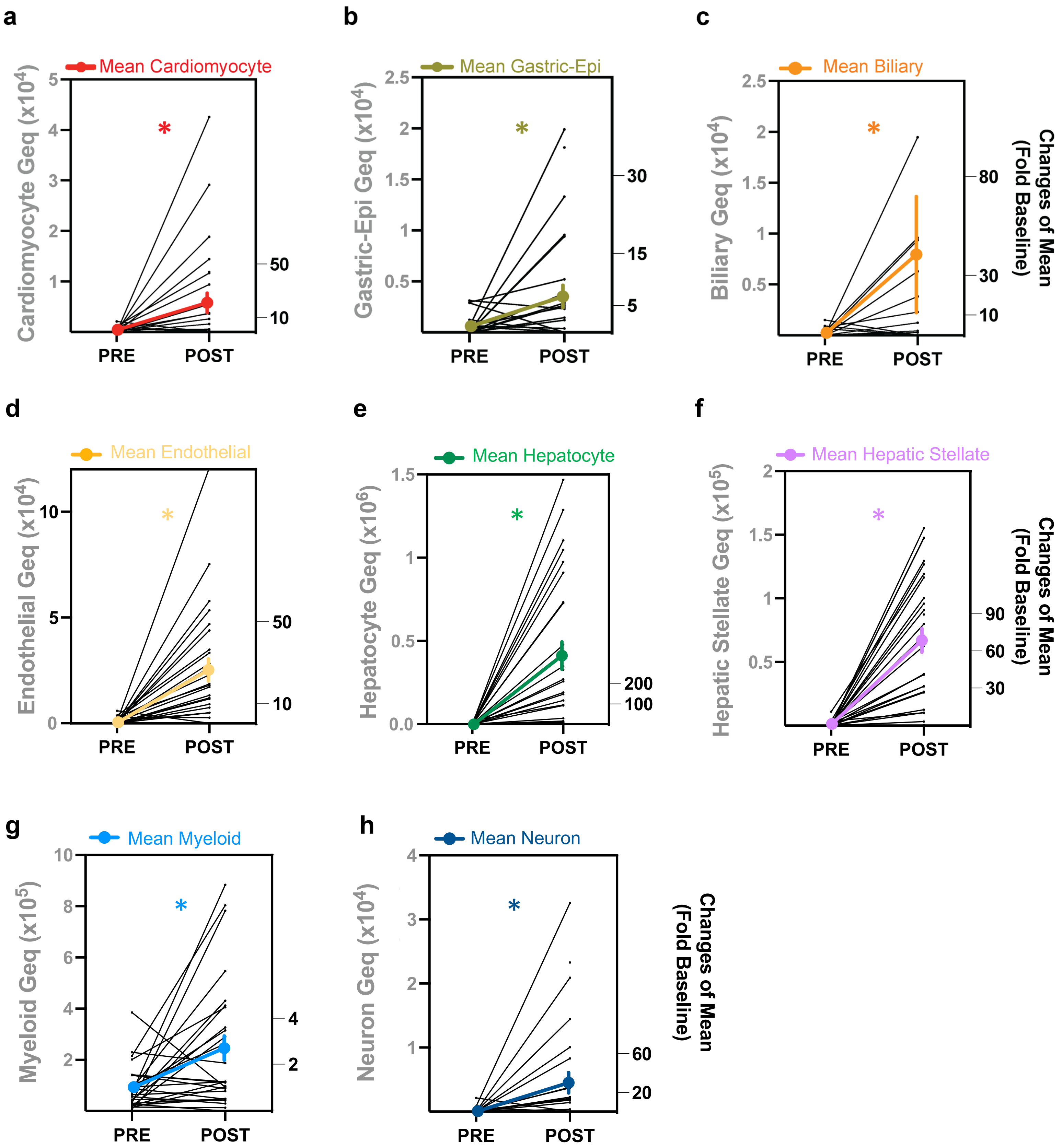

Supplementary Figure 5. Expanded liver cell-type-specific DNA methylation atlases inform origins of cellular damage after liver transplant. a-h, Cardiomyocyte, gastric-epithelial, biliary-epithelial, endothelial, hepatocyte, hepatic stellate, myeloid, and neuron cfDNA (in Geq/mL) in serum samples collected pre-transplant (PRE) or post-reperfusion (POST) on day of surgery (POD0). Mean ± SEM fold change relative to pre-transplant levels is shown in bold. Wilcoxon matched-pairs signed rank test was used for comparison amongst groups (n=28 patients, two-sided). *$p<0.05$; cardiomyocyte $p=0.0027$, gastric-epithelial $p=0.015$, biliary $p=0.0250$, endothelial $p=0.0001$, hepatocyte $p=0.0001$, hepatic stellate $p=0.0001$, myeloid $p=0.0187$, neuron $p=0.0017$.

Supplementary Figure 6

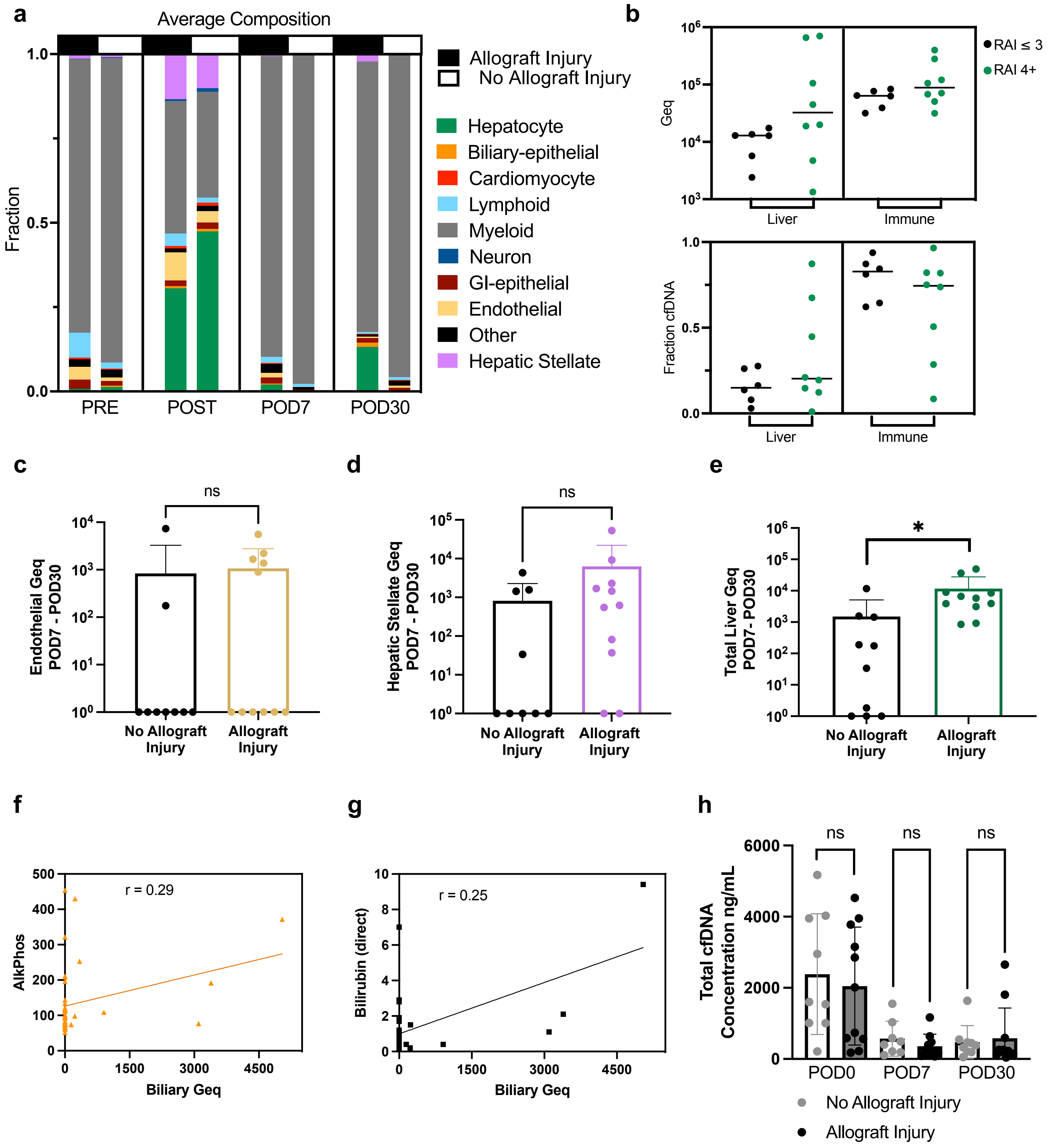

Supplementary Figure 6. Cell-free DNA composition changes after transplant in patients with graft acceptance or injury. a-e, Serum samples from 20 liver transplant patients collected pre-transplant (PRE), post-reperfusion (POD0), post-operative day 7 and 30 (POD7, POD30). By 6 months post-transplant 9 patients showed graft acceptance and 11 patients graft injury. a, Average cfDNA composition estimated from fragment-level deconvolution of serum samples. b, Association of Banff Rejection Activity Index (RAI) lesion grading of liver biopsies at time of clinical diagnosis of allograft injury with liver- and immune-derived cfDNA in the circulation. c, Average of endothelial cfDNA on POD7 and POD30 (Mann-Whitney test, two-sided, ns $p=0.3359$). d, Average of hepatic stellate cfDNA on POD7 and POD30 (Mann-Whitney test, two-sided, ns $p=0.1222$). e, Average of total liver cfDNA (hepatocyte, biliary-epithelial, liver-endothelial and hepatic stellate) on POD7 and POD30 (Mann-Whitney test, two-sided, $p=0.002$). f-g, Correlation of biliary cfDNA (biliary-small-ductal + biliary-large-ductal epithelial) with alkaline phosphatase (ALP) serum levels (f; Spearman $r = 0.29$; two-sided, ns $p=0.0736$) or with serum bilirubin levels (g; Spearman $r = 0.25$; two-sided, ns $p=0.168$). h, Total concentration of cfDNA isolated from patient serum. Individual values and mean ± SD at each timepoint, grouped by outcome. (Mann-Whitney test, two-sided, ns $p>0.05$).

Supplementary Figure 7

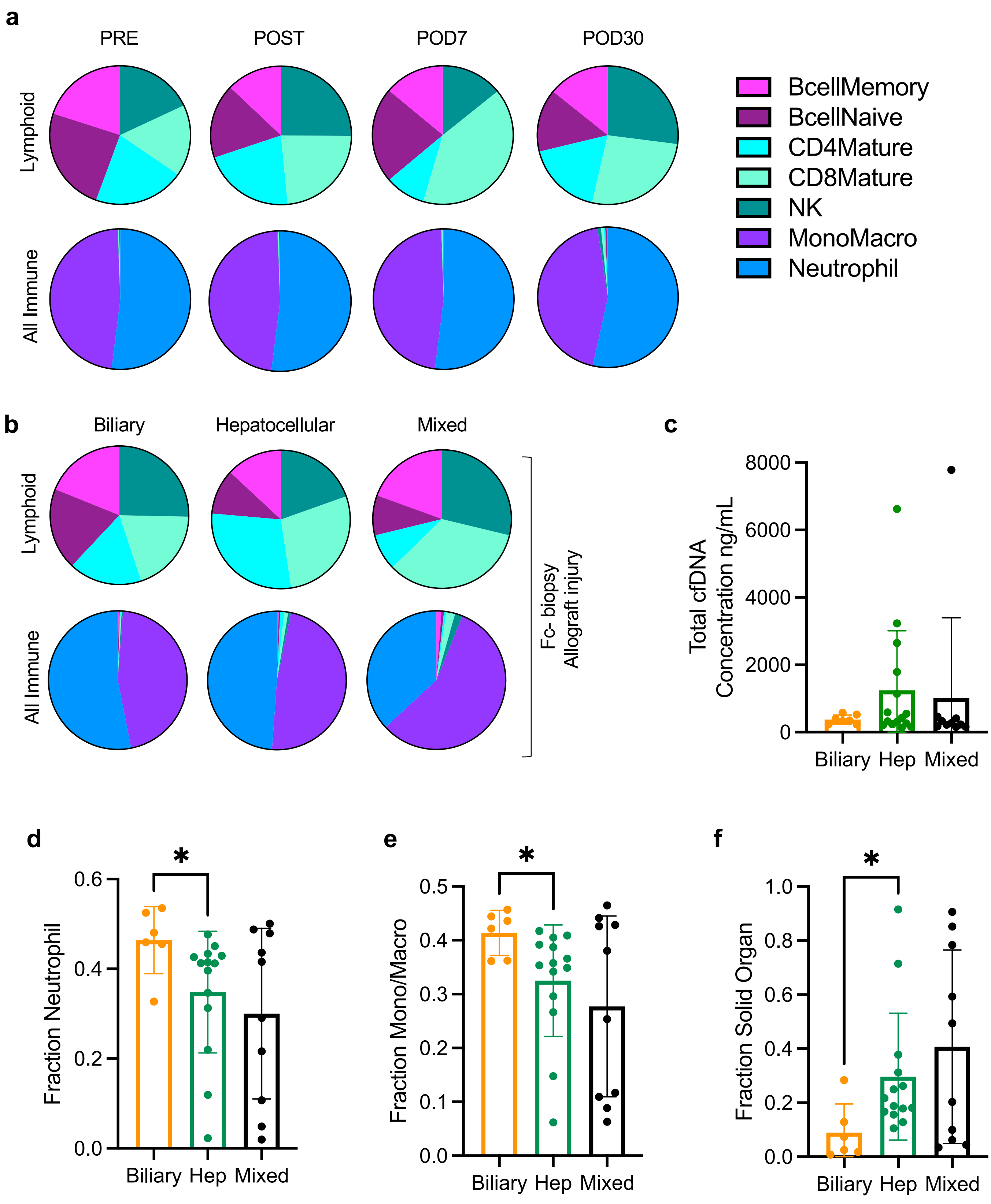

Supplementary Figure 7. Immune cell subset and cell-free DNA composition changes after transplant. a, Average cellular origins of immune cell subsets in cfDNA from serial serum samples from liver transplant patients collected pre-transplant and post-reperfusion on post-operative day 0 (POD0), POD7, and POD30. b, Average cellular origins of immune cell subsets in cfDNA from serum samples collected at the time of for-cause liver biopsies (FC-bx) to diagnose allograft injury (classified by injury patterns observed in biopsies). All biopsies were taken within 1 year of liver transplant and samples are representative of 24 patients (n=30 samples). a-b, (Top) Average proportion of lymphoid immune cell subsets; (Bottom) Average proportion of all immune cell subsets. c, Total concentration of cfDNA isolated from patient serum. Individual values and mean ± SD at each timepoint, grouped by etiology of allograft injury. d, Fraction of neutrophil cfDNA in serum samples with hepatocellular or mixed hepatobiliary injury compared to biliary injury alone. e, Fraction of Monocyte/Macrophage cfDNA in serum samples with hepatocellular or mixed hepatobiliary injury compared to biliary injury alone. f, Fraction of cfDNA from all solid-organ cell-types in serum samples with hepatocellular or mixed hepatobiliary injury compared to biliary injury alone. c-f, Serum samples classified as n=14 hepatocellular, n=10 mixed hepatobiliary, and n=6 biliary etiologies of allograft injury (Mann-Whitney test, two-sided, *$p<0.05$; ns $p>0.05$).

Supplementary Figure 8

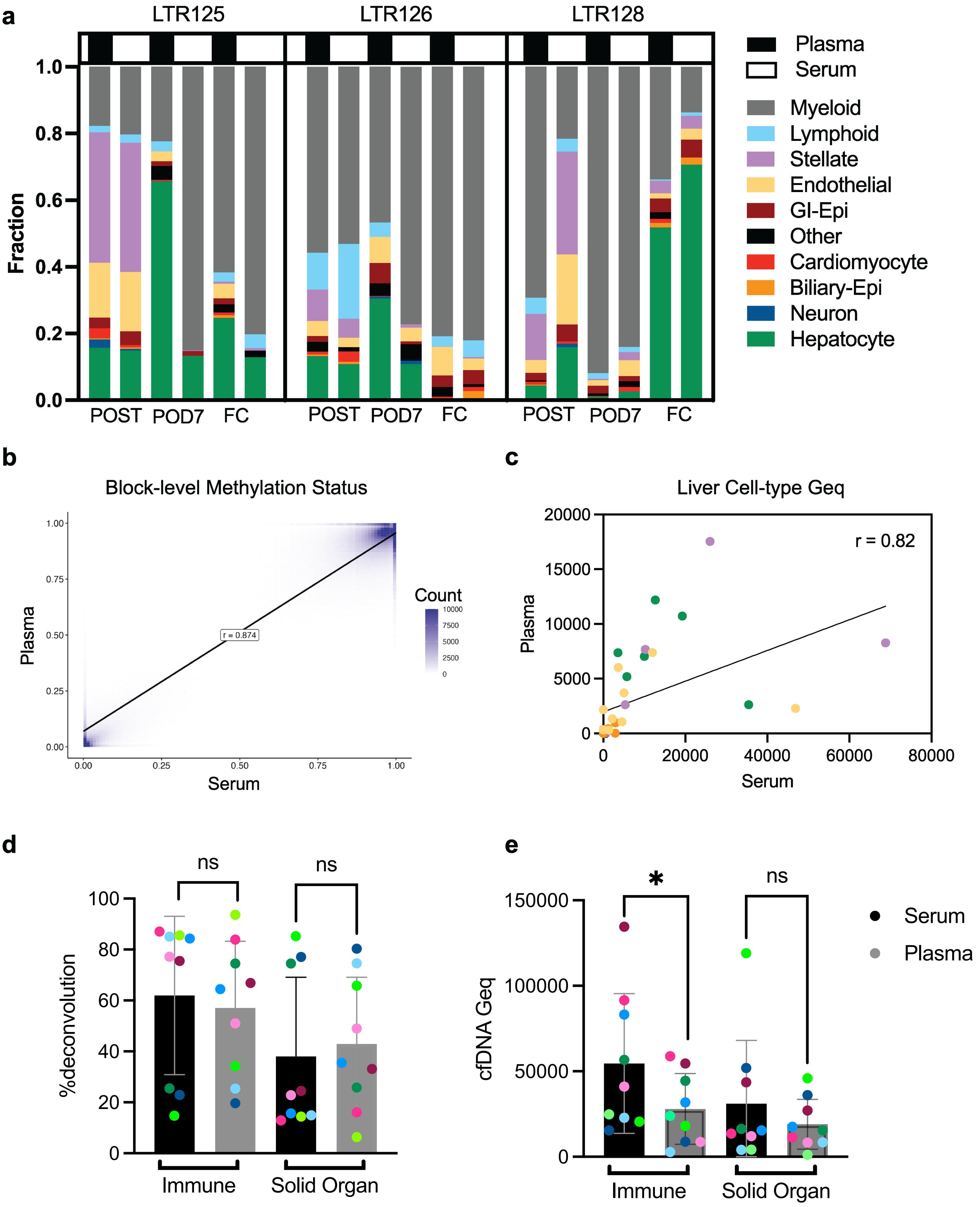

Supplementary Figure 8. Comparison of methylation status and cellular origins of cfDNA isolated from serum and plasma of liver transplant patients. a, Cellular origins of cfDNA fragments in paired serum and plasma samples (n=3 patients). b, Density heatmap comparing methylation status across blocks in cfDNA isolated from paired human serum and plasma (n=9 paired samples). Methylation status is represented by M-values (Logit transformation of β-values) that have normal distribution. Methylation levels are highly correlated at the block level (Pearson's r =0.874, two-sided, $p < 0.05$). c, Correlation of predicted liver cell-type Geq in paired serum and plasma samples (Spearman r = 0.82, two-sided, p =0.0001). d, Predicted %Immune versus %Solid Organ derived cfDNA extracted from either serum or plasma. e, Immune and solid organ Geq from cfDNA isolated from serum versus plasma. d,e, Data presented as mean $\pm$ SD. Individual serum-plasma pairs are represented by differently colored dots. Wilcoxon matched-pairs signed rank test was used for comparisons amongst groups (n=9 samples per group, two-sided). NS, $p \geq 0.05$; *$p < 0.05$.

Supplementary Figure 9

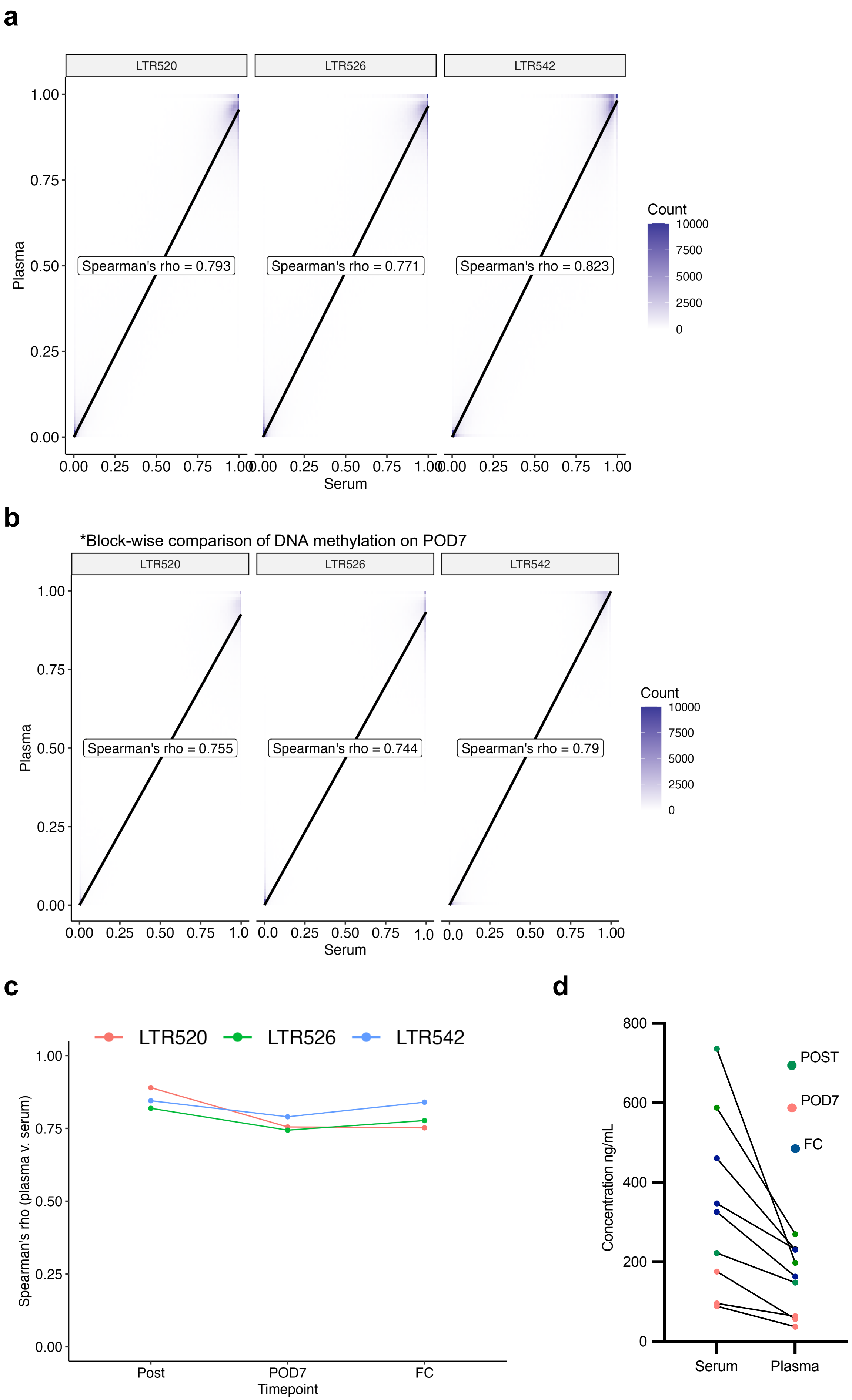

Supplementary Figure 9. Extended pairwise comparison of methylation status and concentration of cfDNA isolated from serum and plasma of liver transplant patients. a, Density heatmap comparing methylation status across blocks in cfDNA isolated from paired human serum and plasma (n=3 individuals each with paired serum and plasma at POD0, POD7, and POD30). Methylation levels are highly correlated at the block level (average Spearman's rho =0.795, two-sided, $p < 0.05$). b, Density heatmap comparing methylation status across blocks in cfDNA isolated from paired human serum and plasma on POD7 only. Methylation levels are highly correlated at the block level (average Spearman's rho =0.763, two-sided, $p < 0.05$). c, Correlation of methylation status across blocks in cfDNA isolated from paired human serum and plasma at each timepoint. d, Concentration of cfDNA isolated from paired serum and plasma (ng/mL) at each timepoint.

Supplementary Figure 10

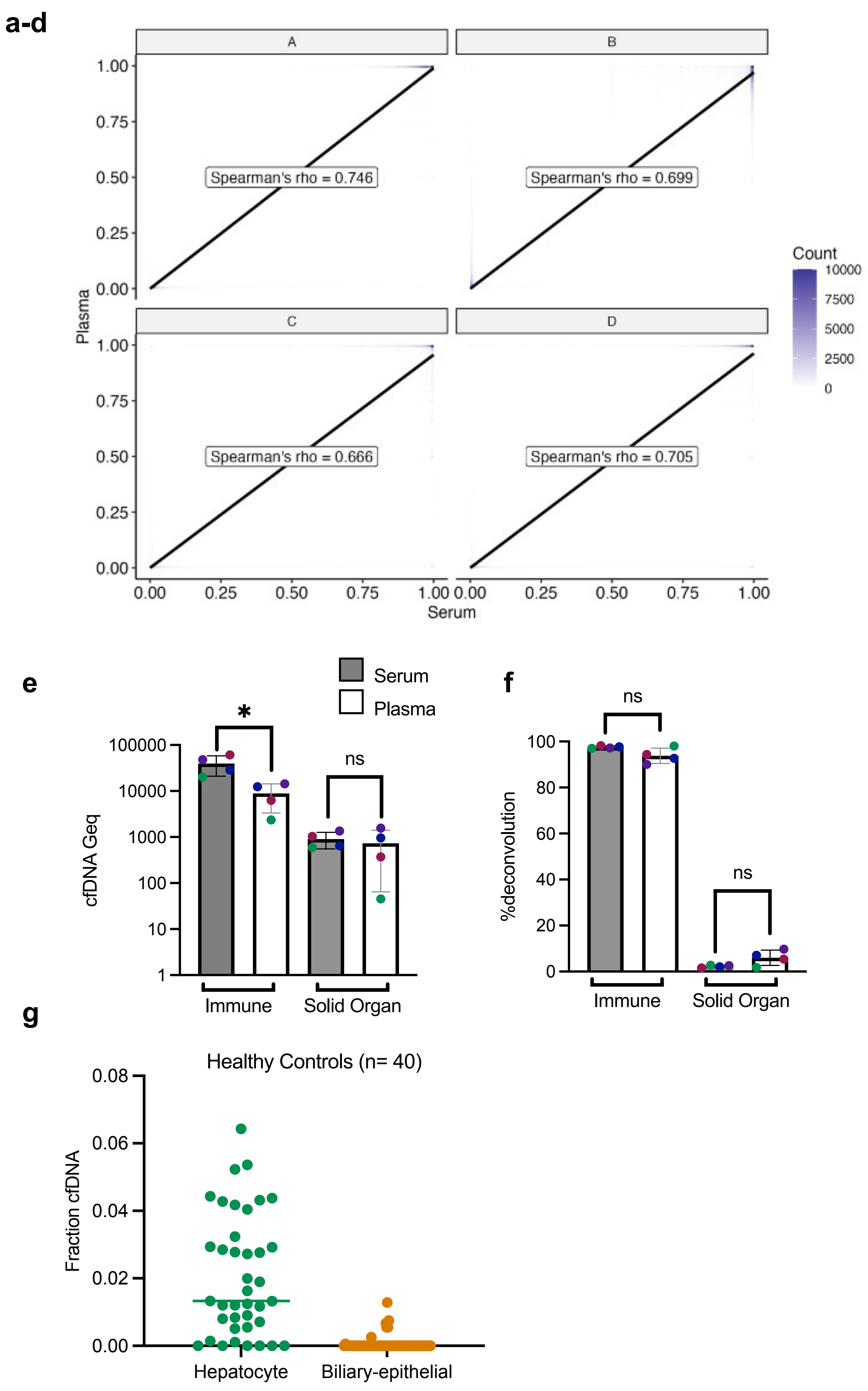

Supplementary Figure 10. Pairwise comparison of methylation status and cellular origins of cfDNA isolated from serum and plasma of healthy human controls. a-d, Density heatmap comparing methylation status across blocks in cfDNA isolated from paired human serum and plasma samples from healthy controls (n=4). e, Immune and solid organ Geq from cfDNA isolated from serum versus plasma. f, Predicted %immune versus %solid-organ derived cfDNA extracted from either serum or plasma. a-f, Methylation data from paired serum and plasma samples from healthy controls reanalyzed from GSE200187. e-f, Data presented as mean ± SD. Individual serum-plasma pairs are represented by differently colored dots. Wilcoxon matched-pairs signed rank test was used for comparisons amongst groups (n=4 samples per group, two-sided, NS, $p \geq 0.05$; $^*p < 0.05$). g, Predicted %hepatocyte versus %biliary-epithelial derived cfDNA extracted from healthy controls (n=40). Data was obtained and reanalyzed from GSE200187, GSE186458, and phs000846.

Supplementary Figure 11

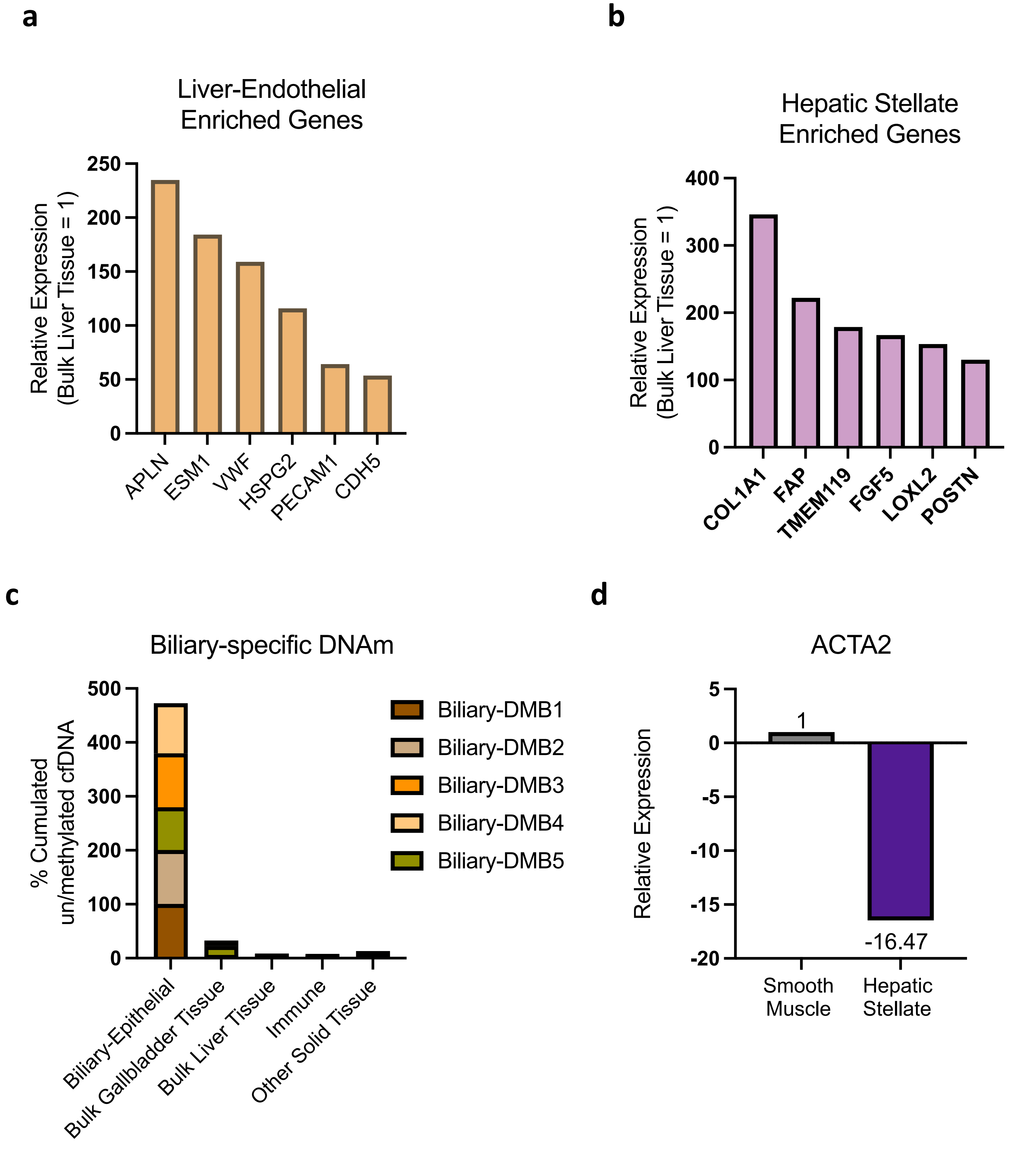

Supplementary Figure 11. Enrichment and purity estimates of sorted cell populations for DNA methylation and RNA-sequencing analysis. a, Liver-endothelial enriched gene expression relative to bulk liver tissue. b, Hepatic-stellate enriched gene expression relative to bulk liver tissue. c, Validation of biliary epithelial cells was done using DNA methylation at previously published regions with specificity to biliary tissues. Proportion of fully methylated or unmethylated fragments was assessed at the following regions (chr2:232262210-232263058 (B3GNT7); chr2:232263147-232263382 (B3GNT7); chr11:1252410-1252491 (MUC5B); chr19:55741530-55741921 (TMEM86B); chr16:58077527-58077828 (MMP15) (DOI: 10.1038/s41586-022-05580-6) d, Fold-expression of ACTA2 (smooth muscle alpha-2 actin) in smooth muscle relative to purified hepatic stellate cell populations. a-d, Expression data were generated from paired RNA-sequencing of the same liver-endothelial and hepatic stellate cell populations used to generate methylation reference data. Bulk liver RNA expression was averaged from 226 bulk liver tissues re-analyzed from GTEx (https://gtexportal.org/home/downloads/adult-gtex/bulk_tissue_expression). Smooth muscle RNA expression data was re-analyzed from the Human Protein Atlas (https://www.proteinatlas.org/download/rna_single_cell_type.tsv).

Supplementary Figure 12

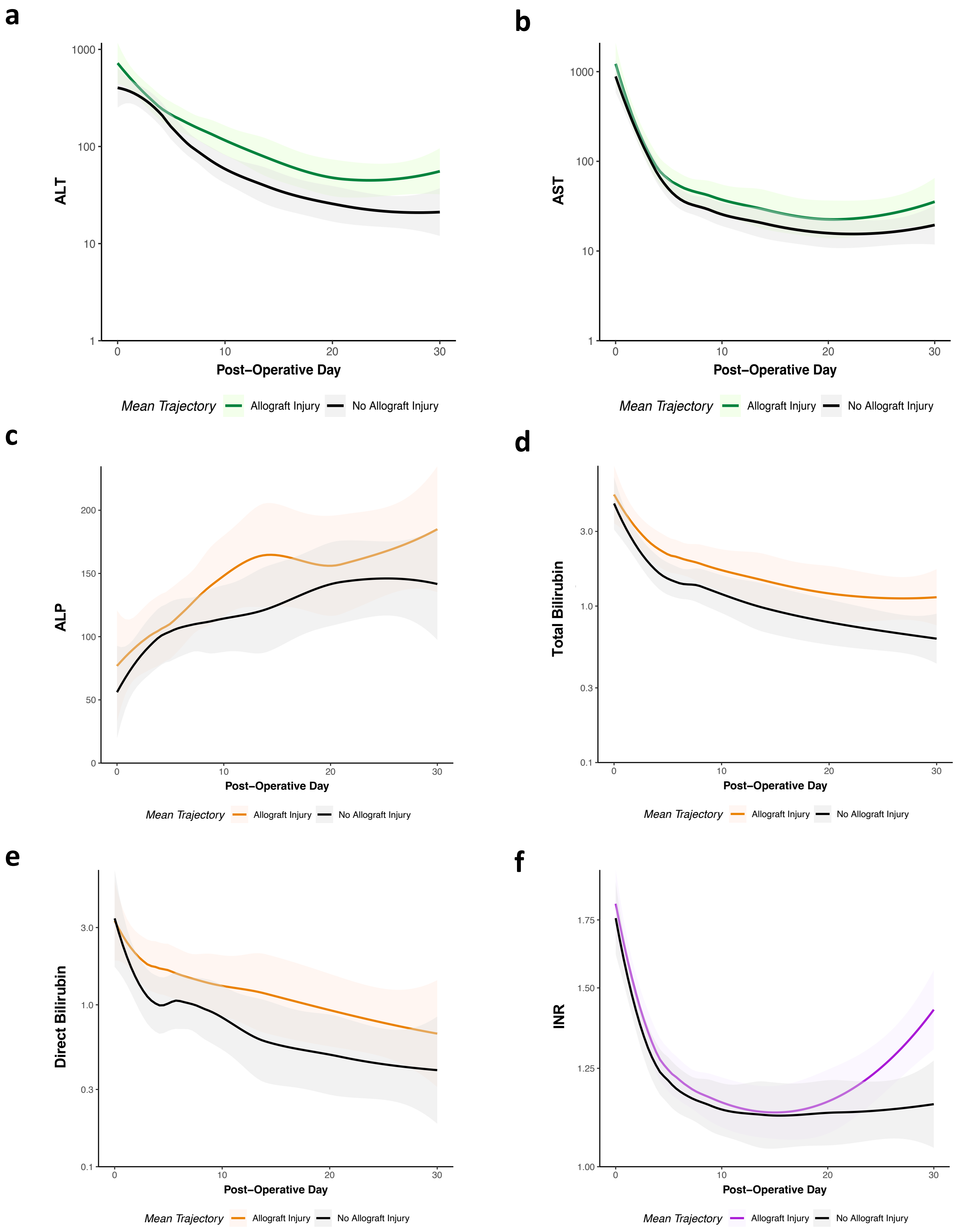

Supplementary Figure 12. Trends in liver function test values over time in patients with and without allograft injury following liver transplantation. a-f, Relationship between time and ALT (alanine aminotransferase), AST (aspartate aminotransferase), ALP (alkaline phosphatase), Total bilirubin, Direct Bilirubin, and INR levels measured at multiple time points post-transplant. Patients were stratified based on the presence or absence of allograft injury, determined by histopathological analysis of liver tissue biopsies within the first-year post-transplant. The solid curve represents the locally weighted regression smoothing (LOESS) trend, highlighting the mean of the fitted values. The shaded region indicates the 95% confidence interval for the LOESS fit. Colored lines indicate patients with allograft injury, and black lines represent those without allograft injury. Wilcoxon rank-sum tests were performed at each timepoint to assess differences in values between the two groups. Wilcoxon rank-sum tests were also performed to assess differences in the mean rate of change in values between the two groups. No statistically significant differences were observed at any timepoint (two-sided, $p > 0.05$).

Supplementary Note 1. Comparison of methylation status in cfDNA isolated from serum and plasma samples.

Paired plasma and serum samples were collected from three liver transplant patients at serial timepoints to compare results across sample preparations. Paired plasma and serum samples from healthy controls were reanalyzed from GSE200187. We computed the average methylation for each block and sample using wgbstools (--beta_to_table)[1]. Correlation analysis was performed comparing the methylation status at the block level between the paired plasma and serum samples (Supplementary Figs. 8, 9, and 10). Deconvolution analysis was performed to compare predicted cell-type proportions. While plasma is generated from whole blood collected in tubes treated with anticoagulant, serum is obtained after allowing blood to clot for 30 minutes at room temperature and then centrifuging the samples to remove the cellular component[2,3]. Cellular components significantly increase in serum samples that sit longer than 60 minutes; however, adherence to standard operating procedures for preparation of serum and plasma have been found to greatly reduce such contamination and sources of error[4]. We took extra steps to address these concerns by ensuring timely processing of blood samples and performing an additional bead purification after cfDNA isolation to remove high-molecular weight DNA derived from blood cell lysis.

Supplementary Note 2. Classification of serum samples corresponding to time of for-cause liver biopsy (FC-bx) to diagnose allograft injury.

Serum samples from 24 liver transplant patients (n = 30 serum samples) were taken at the time of for-cause liver biopsy (FC-bx) to diagnose allograft injury. Patients were classified as having hepatocellular (n=14), biliary (n=6), or mixed hepatobiliary (n=10) etiologies of allograft injury from histopathological analysis of the liver biopsy tissues annotated by a pathologist. The following were defined as clinical etiologies of hepatocellular injury: Acute cellular rejection (ACR, rejection activity index, RAI 3+), recurrence of primary hepatic disease (including recurrence of viral hepatitis (HBV or HCV), autoimmune hepatitis, or (non)-alcoholic steatohepatitis in the transplanted organ), drug-induced hepatotoxicity, ischemia-reperfusion injury (IRI) leading to ischemic hepatitis. The following were defined as clinical etiologies of biliary injury: anastomotic and non-anastomotic biliary strictures, ascending cholangitis, ischemic cholangiopathy, recurrence of primary biliary disease (including primary sclerosing cholangitis (PSC) among others), and septic cholestasis[5–9]. Mixed hepatobiliary forms of allograft injury were characterized by diagnosis of one or more hepatocellular and one or more biliary forms of allograft injury at the same timepoint.

Liver allograft injury was assessed using standardized histological criteria based on established classifications. Acute Cellular Rejection (ACR) was characterized by portal, ductal, and endothelial inflammation, with severity graded as mild, moderate, or severe based on the extent of damage[10]. Antibody-Mediated Rejection (AMR) was identified using a combination of histological features, including microvascular inflammation, C4d staining in portal or sinusoidal vessels, and evidence of donor-specific antibodies

(DSAs)[11]. Ischemic hepatitis was diagnosed by the presence of centrilobular necrosis and minimal inflammatory infiltrates, consistent with reduced blood flow to the liver[12]. Biliary complications were evaluated based on characteristic findings. Cholangitis was identified by neutrophilic infiltrates in bile ducts, while biliary strictures and bile leaks were associated with ductal narrowing, bile duct proliferation, cholestasis, and evidence of bile extravasation[13,14].

Supplementary Note 3. Reference DNA methylation data from healthy tissues and cells.

Availability of previously published and publicly available WGBS data from healthy cell-types and tissues used in this paper are described in Supplementary Data 2. Controlled access to reference WGBS data from normal human tissues and cell types were requested from public consortia participating in the International Human Epigenome Consortium (IHEC)[15] and upon approval downloaded from the European Genome-Phenome Archive (EGA), Japanese Genotype-phenotype Archive (JGA), database of Genotypes and Phenotypes (dbGAP), and ENCODE portal data repositories[16–20]. Reference WGBS data were also downloaded from selected GEO and SRA datasets[21–28]. Reference WGBS data were analyzed as previously described.

Segmentation and clustering analysis

We segmented the genome into blocks of homogenous methylation as previously described [27]. In brief, a Dynamic Programming segmentation algorithm was used to divide the genome into continuous genomic regions (blocks) showing homogenous methylation levels across multiple CpGs for each sample. We applied the segmentation algorithm to over 450 human reference WGBS methylomes and retained 364,268 blocks covered by the hybridization capture panel used in the analysis of cfDNA (probed regions span 80Mb (~20% of CpGs) on the capture panel). The top 10% most variable methylation blocks containing at least three CpG sites and coverage across 90% of samples were selected, irrespective of sample cell-type group. We computed the average methylation for each block and sample using wgbstools (--beta_to_table). Dimensional reduction was performed on the selected blocks using the UMAP package (V 0.2.8.2.0)[29]. Default UMAP

parameters were used (15 neighbors, 2 components, Euclidean metric, and a minimum distance of 0.1).

Identification of cell-type specific methylation blocks

Tissue and cell-type specific methylation blocks were identified from reference WGBS as previously described[28]. We performed a one-vs-all comparison to identify differentially methylated blocks unique for each group. All cell-type-specific blocks contained a minimum of three CpG sites, with lengths of less than 2kb and at least 10 observations. In brief, we calculated the average methylation per block/sample, as the ratio of methylated CpG observations across all sequenced reads from that block. Differential blocks were sorted by the margin of separation, termed "delta beta", defined as the minimal difference between the average methylation in any sample from the target group vs all other samples. Then, we computed the "soft margin" between target samples and background samples, allowing for some outliers using percentiles. For all hypomethylation markers we calculated the difference between the 80th percentile of the methylation status in the target group (--target.quant 0.2) and the 10th percentile of the methylation status in the background group (--bg.quant 0.1). Conversely, for all hypermethylated markers we calculated the difference between the 20th percentile methylation status in the target group and the 90th percentile in the background group. We selected blocks with a margin ≥ 0.4 for all cell-type groups. Blocks with a (-) direction are hypomethylated and (+) direction are hypermethylated, defined as a direction of methylation in the target cell-type relative to all other tissues and cell-types included in the atlas. We also used a magnitude threshold where all hypomethylated blocks have an Average Methylation Fraction (AMF) <0.5 and hypermethylated blocks have an AMF

>0.5. However, the vast majority of cell-type specific differentially methylated blocks are much more diverged (mean AMF hypo = <10% methylation and mean AMF hyper = >80%).

Biliary epithelial samples were separated into two groups of epithelial populations, samples isolated from intrahepatic ducts and the gallbladder (biliary-small-ductal) compared to samples isolated from the larger main hepatic, common bile and pancreatic ducts (biliary-large-ductal). DMBs were identified for each biliary epithelial cell population. Estimated cell proportions from biliary-small-ductal and biliary-large-ductal epithelial cell proportions were combined to reflect the total biliary cfDNA from deconvolution analysis of serum samples from liver transplant patients. Endothelial samples were combined to identify common endothelial DMBs across all tissues. However, we ensured that methylation status was conserved in liver sinusoidal endothelial methylomes for all identified common endothelial-specific methylation blocks. Liver-resident immune (CD14+,CD11b+,CD68+) samples were grouped with other myeloid immune samples when identifying markers for deconvolution of cfDNA fragments from liver transplant samples. Only two liver-resident immune cell-specific DMBs were identified when these samples were considered as a separate group at the same thresholds that other cell-type-specific DMBs were identified in the atlas. However, extended liver-resident immune cell markers were identified with relaxed thresholds (--margin 0.3, --target.quant 0.2, --bg.quant 0.2) to use for characterization of liver cell-specific epigenetic data. Chromatin accessibility and histone modification data in Figure 3A-C is plotted using identified biliary-small-ductal epithelial, common endothelial, and extended liver-resident immune cell-specific DMBs (Supplementary Data 3 and 7). There were also a reduced number of

highly specific DMBs identified for peripheral immune cell subsets relative to all other cell-types. The top 25 DMBs specific to peripheral immune cell subsets (--margin 0.4, --target.quant 0.2, --bg.quant 0.1) were used and deconvolution estimates were fit into the proportions for total myeloid and total lymphoid immune cell estimates using supergroups of neutrophils, monocytes, and macrophages to form a combined myeloid group and mature B cell, naïve B cell, CD4 T cell, CD8 T cell and NK cell to form a combined lymphoid group. The estimated deconvolution results of immune cell subsets are provided in Supplementary Fig. 7 and Supplementary Data 9.

References

1. Loyfer & Kaplan. *Wgbstools: A Computational Suite for DNA Methylation Sequencing Data Representation, Visualization, and Analysis.* (2023).

2. Kerachian, M. A., Azghandi, M., Mozaffari-Jovin, S. & Thierry, A. R. Guidelines for pre-analytical conditions for assessing the methylation of circulating cell-free DNA. *Clin Epigenetics* **13**, 193 (2021).

3. Holdenrieder, S. *et al.* Cell-Free DNA in Serum and Plasma: Comparison of ELISA and Quantitative PCR. *Clin Chem* **51**, 1544–1546 (2005).

4. Tuck, M. K. *et al.* Standard Operating Procedures for Serum and Plasma Collection: Early Detection Research Network Consensus Statement Standard Operating Procedure Integration Working Group. *J Proteome Res* **8**, 113–117 (2009).

5. Seehofer, D., Eurich, D., Veltzke-Schlieker, W. & Neuhaus, P. Biliary Complications After Liver Transplantation: Old Problems and New Challenges. *Am. J. Transplant.* **13**, 253–265 (2013).

6. Agostini, C. *et al.* Complications in Post-Liver Transplant Patients. *J. Clin. Med.* **12**, 6173 (2023).

7. Craig, E. V. & Heller, M. T. Complications of liver transplant. *Abdom. Radiol.* **46**, 43–67 (2021).

8. Civantos, D. V. P. *et al.* Liver Pathology. (2021) doi:10.5772/intechopen.89094.

9. Fasullo, M., Patel, M., Khanna, L. & Shah, T. Post-transplant biliary complications: advances in pathophysiology, diagnosis, and treatment. *BMJ Open Gastroenterol.* **9**, e000778 (2022).

10. Demetris, A. J. *et al.* Banff schema for grading liver allograft rejection: An international consensus document. *Hepatology* **25**, 658–663 (1997).

11. Lee, B. T., Fiel, M. I. & Schiano, T. D. Antibody-mediated rejection of the liver allograft: An update and a clinico-pathological perspective. *J. Hepatol.* **75**, 1203–1216 (2021).

12. CIOBANU, A. O. & GHERASIM, L. Ischemic Hepatitis – Intercorrelated Pathology. *MAEDICA a J. Clin. Med.* **13**, 5–11 (2018).

13. Gonzalez, R. S. & Washington, K. Primary Biliary Cholangitis and Autoimmune Hepatitis. *Surg. Pathol. Clin.* **11**, 329–349 (2018).

14. Hiramatsu, K. *et al.* Proposal of a new staging and grading system of the liver for primary biliary cirrhosis. *Histopathology* **49**, 466–478 (2006).

15. Bujold, D. *et al.* The International Human Epigenome Consortium Data Portal. *Cell Syst* **3**, 496-499.e2 (2016).

16. Dunham, I. *et al.* An integrated encyclopedia of DNA elements in the human genome. *Nature* **489**, 57–74 (2012).

17. Consortium, T. E. P. *et al.* Expanded encyclopaedias of DNA elements in the human and mouse genomes. *Nature* **583**, 699–710 (2020).

18. Consortium, R. E. *et al.* Integrative analysis of 111 reference human epigenomes. *Nature* **518**, 317 (2015).

19. Fernández, J. M. *et al.* The BLUEPRINT Data Analysis Portal. *Cell Syst* **3**, 491-495.e5 (2016).

20. Davis, C. A. *et al.* The Encyclopedia of DNA elements (ENCODE): data portal update. *Nucleic Acids Res* **46**, gkx1081- (2017).

21. Pidsley, R. *et al.* Critical evaluation of the Illumina MethylationEPIC BeadChip microarray for whole-genome DNA methylation profiling. *Genome Biol* **17**, 208 (2016).

22. Gilsbach, R. *et al.* Distinct epigenetic programs regulate cardiac myocyte development and disease in the human heart in vivo. *Nat Commun* **9**, 391 (2018).

23. Farlik, M. *et al.* DNA Methylation Dynamics of Human Hematopoietic Stem Cell Differentiation. *Cell Stem Cell* **19**, 808–822 (2016).

24. Dong, X. *et al.* Global, integrated analysis of methylomes and transcriptomes from laser capture microdissected bronchial and alveolar cells in human lung. *Epigenetics* **13**, 1–22 (2018).

25. Jamil, M. A. *et al.* Molecular Analysis of Fetal and Adult Primary Human Liver Sinusoidal Endothelial Cells: A Comparison to Other Endothelial Cells. *Int J Mol Sci* **21**, 7776 (2020).

26. Cheng, T. H. T. *et al.* Noninvasive Detection of Bladder Cancer by Shallow-Depth Genome-Wide Bisulfite Sequencing of Urinary Cell-Free DNA for Methylation and Copy Number Profiling. *Clin Chem* **65**, 927–936 (2019).

27. Loyfer, N. *et al.* A DNA methylation atlas of normal human cell types. *Nature* **613**, 355–364 (2023).

28. McNamara, M. E. *et al.* Circulating cell-free methylated DNA reveals tissue-specific, cellular damage from radiation treatment. *JCI Insight* **8**, e156529 (2023).

29. McInnes, L., Healy, J. & Melville, J. UMAP: Uniform Manifold Approximation and Projection for Dimension Reduction. *Arxiv* (2018).
