# Supplementary material for: Circulating cell-free DNA methylation patterns indicate cellular sources of allograft injury after liver transplant
Source: Nat Commun. 2025 Jun 17;16:5310. doi: 10.1038/s41467-025-60507-9 (PMC12174327; doi:10.1038/s41467-025-60507-9)
Supplement: Supplementary file 2 — Description of Additional Supplementary Files [file 41467_2025_60507_MOESM2_ESM.docx]

**Description of Additional Supplementary Files**

**Supplementary Data 1.** Characteristics of liver transplant patients enrolled in this study.

**Supplementary Data 2.** Human reference methylation data from healthy tissues and cell types.

**Supplementary Data 3.** Identified human liver cell-type-specific methylation blocks (margin 0.4;bg.quant 0.1;tg.quant 0.2). Annotation was performed using Homer. The margin of separation represents the delta-beta (maximum higher – minimum lower) across all samples. Blocks with a (-) direction are hypomethylated and (+) direction are hypermethylated. AMF (average methylation fraction) indicated as a fraction.

**Supplementary Data 4.** Significantly enriched biological pathways for genes associated with differential methylation in each cell-type. Correction for multiple hypothesis comparisons was performed using the Benjamini-Hochberg (B-H) method corrected p-value to control the false discovery rate (FDR) from multiple pathways being tested against each gene-set (one-sided).

**Supplementary Data 5.** Significantly enriched motifs from transcription factor binding site analysis (using HOMER findMotifsGenome.pl function). Shown are binomial pvalues (one-sided). (a) Enriched motifs for individual liver cell-type-specific hypomethylated blocks (b) Enriched motifs for all hypomethylated DMBs in all liver celltypes combined. (c) Enriched motifs for all hypermethylated DMBs in all liver cell-types combined.

**Supplementary Data 6.** (a) Liver transplant serial cfDNA sample concentrations and predicted cell-type proportions from fragment-level deconvolution analysis (n=28 patients; n=100 samples). (b) Liver transplant phenotype-matched cfDNA sample concentrations and predicted cell-type proportions at FC-bx from fragment-level deconvolution analysis (additional n=16 patients; n=30 samples).

**Supplementary Data 7.** Extended liver-resident immune cell-type-specific methylation blocks (margin 0.3;bg.quant 0.2;tg.quant 0.2). Annotation was performed using Homer. The margin of separation represents the delta-beta (maximum higher – minimum lower) across all samples. Blocks with a (-) direction are hypomethylated and (+) direction are hypermethylated. AMF (average methylation fraction) indicated as a fraction.

**Supplementary Data 8.** (a) Liver transplant serial cfDNA sample concentrations and predicted cell-type Geq from fragment-level deconvolution analysis (n=28 patients; n=100 samples). (b) Liver transplant phenotype-matched cfDNA sample concentrations and predicted cell-type Geq at FC-bx from fragment-level deconvolution analysis (additional n=16 patients; n=30 samples).

**Supplementary Data 9.** (a) Liver transplant serial cfDNA sample concentrations and predicted immune subset cell-type proportions from fragment-level deconvolution analysis (n=28 patients; n=100 samples). (b) Liver transplant phenotype-matched cfDNA sample concentrations and predicted immune subset cell-type proportions at FC-bx from fragment-level deconvolution analysis (additional n=16 patients; n=30 samples).
